# Segmentation and Modelling of the Nuclear Envelope of HeLa Cells Imaged with Serial Block Face Scanning Electron Microscopy †

**DOI:** 10.3390/jimaging5090075

**Published:** 2019-09-12

**Authors:** Cefa Karabağ, Martin L. Jones, Christopher J. Peddie, Anne E. Weston, Lucy M. Collinson, Constantino Carlos Reyes-Aldasoro

**Affiliations:** 1 Department of Electrical and Electronic Engineering, Research Centre for Biomedical Engineering, School of Mathematics, Computer Science and Engineering, City, University of London, London EC1V 0HB, UK; 2 Electron Microscopy Science Technology Platform, The Francis Crick Institute, London NW1 1AT, UK; martin.jones@crick.ac.uk (M.L.J.); christopher.peddie@crick.ac.uk (C.J.P.); anne.weston@crick.ac.uk (A.E.W.); lucy.collinson@crick.ac.uk (L.M.C.)

**Keywords:** segmentation, HeLa cells, active contours, Hausdorff distance, Jaccard index

## Abstract

This paper describes an unsupervised algorithm, which segments the nuclear envelope of HeLa cells imaged by Serial Block Face Scanning Electron Microscopy. The algorithm exploits the variations of pixel intensity in different cellular regions by calculating edges, which are then used to generate superpixels. The superpixels are morphologically processed and those that correspond to the nuclear region are selected through the analysis of size, position, and correspondence with regions detected in neighbouring slices. The nuclear envelope is segmented from the nuclear region. The three-dimensional segmented nuclear envelope is then modelled against a spheroid to create a two-dimensional (2D) surface. The 2D surface summarises the complex 3D shape of the nuclear envelope and allows the extraction of metrics that may be relevant to characterise the nature of cells. The algorithm was developed and validated on a single cell and tested in six separate cells, each with 300 slices of 2000 × 2000 pixels. Ground truth was available for two of these cells, i.e., 600 hand-segmented slices. The accuracy of the algorithm was evaluated with two similarity metrics: Jaccard Similarity Index and Mean Hausdorff distance. Jaccard values of the first/second segmentation were 93%/90% for the whole cell, and 98%/94% between slices 75 and 225, as the central slices of the nucleus are more regular than those on the extremes. Mean Hausdorff distances were 9/17 pixels for the whole cells and 4/13 pixels for central slices. One slice was processed in approximately 8 s and a whole cell in 40 min. The algorithm outperformed active contours in both accuracy and time.

## 1. Introduction

Cervical cancer is one of the leading causes of death in females [1], and together with breast cancer, it contributes to 4.2% of the global causes of death [2]. In the United Kingdom, around 3000 women are diagnosed with cervical cancer every year, and around 1000 of those die annually [3]. Cancer cell lines and tissue culture studies have been used extensively to understand tumour biology and facilitate drug discovery processes [4,5,6]. The availability of cell lines, together with genetic advances, has contributed to cancer research, which has grown significantly as a proportion of all research in biomedical areas [7]. Perhaps the most important cell line has been the cervical cancer HeLa cells, derived in 1951 [8,9] and widely used as a model for thousands of biological experiments [10,11]. 

Electron Microscopy (EM) is an imaging technique that provides resolving power several orders of magnitude higher than conventional light and fluorescence microscopes. Recent developments in semi-automated image acquisition have prompted a revival in the use of EM, transforming biomedical imaging experiments. Serial blockface scanning EM (SBF SEM) was developed and implemented by Denk and Horstmann [12] to enable the automation of serial imaging of relatively large volumes with nanometer resolution. SBF SEM utilises an ultramicrotome with a diamond knife to cut and discard very thin slices or sections from the top face of the sample. The sample is then raised to the focal plane to be scanned, and this process is repeated sequentially, resulting in a stack of images through the sample volume [13]. With the advent of SBF SEM and other volume electron microscopy techniques [13,14,15], three-dimensional visualisation of specimens with unprecedented detail became possible. However, as previously experienced by the genomics and proteomics communities, technological advances in data acquisition displaced the bottleneck from data generation to data processing [16,17].

Cell segmentation and classification has been an important and challenging problem for many years [18,19,20,21]. It has attracted considerable attention, both in clinical practice and computing research, as the identification of individual cells and the shape of the cell and its parts, like the nuclear envelope (NE), may reveal some conditions of health or disease [22]. The observation of the nucleus is a key element in the study of cancerous cells [23]; however, the organisation of the nucleus itself remains an area largely unexplored [24]. One of the reasons behind this lack of research is due to the high resolution required to reveal cells’ fine structures [25,26], which in turn requires complex algorithms and difficult to obtain ground truth segmentations. However, segmentation and analysis of electron micrographs is particularly challenging for several reasons. First, each image in the stack is large, typically 8000 × 8000 pixels. Each image can be 250 MB in size, so acquisition of serial images can quickly generate gigabytes or terabytes of data. Second, a major bottleneck for any quantitative analysis is the time required for expert interpretation and annotation/segmentation of the images. Segmentation of cells and organelles in electron micrographs is, in many cases, still a manual process [27,28,29], despite intensifying efforts by the community to automate the process. This is largely due to complexity and diversity of features within a single image, many of which have similar grey values, making a clean histogram-based feature extraction unlikely. The success of algorithms designed for feature extraction is highly dependent on the contrast and signal-to-noise ratio of the image, as well as the resolution, the crowding and diversity of features, and the heterogeneity of the appearance of the feature in the image, which is linked to its morphology and orientation in the two-dimensional (2D) slice. These, and other properties of the sample and image, mean that techniques that work well for imaging techniques like immunohistochemistry and light microscopy (e.g., watersheds) [30,31,32,33] do not usually port well to EM. Shallow [34,35] and deep learning methodologies [36,37] are becoming popular for segmentation and classification of image data. However, these techniques require significant computational power, as well as very large training data sets [38,39], which are rarely available in biological electron microscopy and were not available for our specific data sets. There are some commercial environments with sophisticated segmentation tools like Amira^TM^ (Thermo Fisher Scientific, Waltham, MA, USA), IMARIS software (Bitplane, Belfast, UK), and AIVIA (Bellevue, WA, USA) have been used in similar work [40,41,42]. However, the cost of these tools may prevent many scientists from using them, and the development of automated and open-source algorithms is therefore necessary.

Rather than relying on low-throughput expert segmentation to produce training data, an alternative approach is being developed, taking advantage of citizen science, where an army of non-experts [43] are recruited to provide non-expert human annotation, segmentation, or classification through web-based interfaces. In parallel to the work described here, a project called Etch a Cell [44] was created to provide manual segmentation of the NE of HeLa cells. The NE is the membrane that surrounds the chromosomes and partitions them from the rest of the cell contents, thereby forming a protected environment for the genetic material. Almost every cell (red blood cells being a notable exception) has a nucleus bounded by a nuclear envelope, and so it is a primary target for automated segmentation as a reference structure for visualising and spatial positioning of other cell organelles, and as a feature in its own right as its morphology is known to alter in cancer, infection, and in rare genetic disorders such as the laminopathies. For the NE, the delineation of the nuclear envelope through the citizen science portal is producing promising results, but further development is required to quality control, aggregate, and train with these non-expert contributions. Thus, specific processing algorithm for automated analysis and visualisation of cell features in large microscopy data sets are still important [45,46,47,48], both for direct analysis of data and for production of additional training data.

In this paper, an unsupervised image-processing algorithm to segment the NE of HeLa cervical cancer cells in images acquired via SBF SEM is described. The algorithm follows a pipeline of traditional image-processing steps: Low-pass filtering, edge detection, dilations, determination of superpixels, distance transforms, morphological operators, and post-processing to automatically segment the nuclear envelope. First, the algorithm calculates edges that separate regions with pixels of different intensities. The standard Canny algorithm [49] is used, but then the edges are further dilated to connect those edges that may be separated by a few pixels. This could be the case of edges detected due to changes of intensity of the nuclear envelope itself. Next, superpixels are generated by labelling the regions that are not covered by the dilated edges. The superpixel size is not restricted to allow the existence of large superpixels, e.g., those that cover the background. Small superpixels and those in contact with the boundary of the image are discarded by using morphological operators, and the remaining superpixels are smoothed and filled. The selection of the nuclear region requires the analysis of contiguous slices; this follows the process that human operators apply when analysing disjoint nuclear regions. Finally, the algorithm determines the boundary of this nuclear region as the NE. The segmented NE is then modelled against a spheroid to quantitatively analyse the shape of the nucleus.

The algorithm here described assumes the following: (1) There is a single HeLa cell of interest, which may be surrounded by fragments of other cells, but the centre of the cell of interest is located at centre of a three-dimensional (3D) stack of images; (2) the nuclear envelope is darker than the nuclei or its surroundings; and (3) the background is brighter than any cellular structure. The centroid of the cells has been hand located and placed into the centre of the data sets; however, we have extensions of the algorithm to do this automatically (these and other extensions are available in the GitHub pages). The algorithm segments the NE of HeLa cervical cancer cells in approximately 8 s and a whole cell contained within 300 slices in approximately 40 min. Except for the manual selection of the centroid to crop each cell, the algorithm is fully automatic.

A preliminary version of this work was presented at the 22^nd^ Medical Image Understanding and Analysis (MIUA) Conference [50]. This paper describes an extended algorithm, which now considers the following: (i) Detection of disjoint areas of the nucleus by comparing between neighbouring slices—this approach is similar to what a human expert will do by scrolling up and down when performing a manual delineation; (ii) the nuclear envelope of each cancerous HeLa cell was modelled against a spheroid—characteristics of the volumetric shape can be summarised into simple metrics; (iii) sensitivity analyses to optimize the algorithm for the segmentation were performed; (iv) seven different cells were segmented and two of them were compared against their corresponding ground truth; (v) in order to assess the algorithm, Hausdorff distance (HD) was calculated to compare the ground truth with the algorithm segmentation as an alternative to Jaccard index which was performed previously; (vi) active contour segmentation was performed on the same cells and results were compared with the algorithm segmentation; and (vii) an artefact, which was reported in [50], due to the slight shift in the acquisition, was resolved with a pre-alignment. Furthermore, the algorithms developed for this work are shared in an open-source way through GitHub and the data sets through EMPAIR (see Supplementary Materials). It should be noted that a Matlab License is necessary to run the algorithms.

The results obtained with the algorithms presented here may lead to important biological observations of the nature of the cells being analysed, but any practical application or biological relevance are beyond the scope of this paper.

## 2. Materials and Methods

### 2.1. HeLa Cells’ Preparation and Acquisition

**Figure 1:**
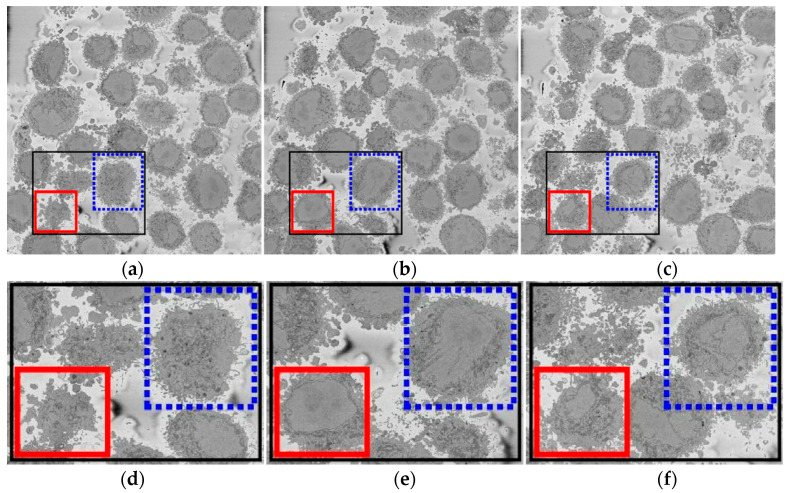
Three representative slices of a three-dimensional (3D) image stack acquired by Serial Block Face Scanning Electron Microscope (SBF SEM) containing numerous HeLa cells. Boxes indicate two regions of interest (ROIs), which contain two of the cells that will be posteriorly segmented. (**a**) A slice on the lower section of the stack; slice 43 of 300. (**b**) A slice on the central section; 118/300. (**c**) A slice on the higher section; 241/300. Black box denotes the ROI that is magnified in (**d**–**f**). Notice the differences in sizes of cell and nuclei in the images. In particular, the nuclei are hardly visible in (**a**), largest in (**b**), and in (**c**) the nucleus in the blue dotted box appears as several disjoint regions surrounded by a darker nuclear envelope (NE).

HeLa cells were prepared for SBF SEM following the method of the National Centre for Microscopy and Imaging Research (NCMIR) [51]. SBF SEM data were collected using a 3View2XP (Gatan, Pleasanton, CA) attached to a Sigma VP SEM (Zeiss, Cambridge). In total, 517 images of 8192 × 8192 pixels were acquired. Voxel size was 10 × 10 × 50 nm with intensity [0—255] (Figure 1). Initially, the data were acquired at higher bit-depth (32 bit or 16 bit) and, after contrast/histogram adjustment, reduced to 8 bit. For this paper, seven individual cells were manually cropped as volumes of interest. For each cell, the centroid was manually selected as the centre of a sub-volume of 300 slices with dimensions (*n_h_*, *n_w_*, *n_d_*) = (2000, 2000, 1) and were saved as single channel TIFF files. Images are openly accessible via the EMPAIR public image database (http://dx.doi.org/10.6019/EMPIAR-10094).

### 2.2. Ground Truth (GT)

Two cells (i.e., 600 slices) were manually segmented to provide a ground truth to assess the accuracy of the algorithm. Each cell was segmented by different persons without knowledge of each other and with different acquisition conditions. The first cell (shown by the red box in Figure 1) was segmented using Amira and a Wacom Cintiq 24HD interactive pen display by one of the authors (A.E.W.) and took around 30 h. The second cell (shown by the blue box in Figure 1) was segmented by another author (C.K.) with a Wacom pen-and-tablet using the MATLAB^®^ roipoly function and took around 47 h. In both cases, in order to determine whether disjoint regions belong to the nucleus, the user scrolled up and down through neighbouring slices to check connectivity of the regions.

### 2.3. Automatic Segmentation of the Nuclear Envelope Algorithm

**Figure 2:**
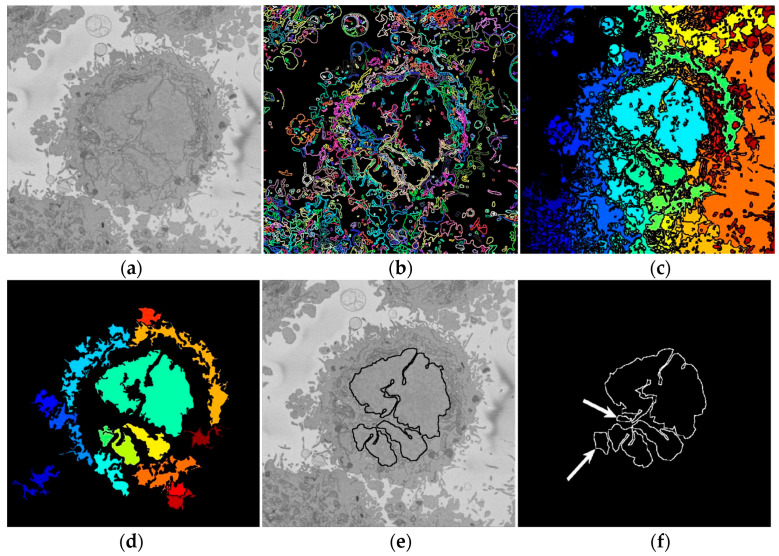
Intermediate steps of the segmentation algorithm. (**a**) Cropped region around one HeLa cell (dotted blue box in Figure 1c,f), surrounded by resin (background) and edges of other cells. This image was low-pass filtered. (**b**) Edges detected by Canny algorithm. The edges were further dilated to connect those edges that may belong to the nuclear envelope (NE) but were disjoint due to the variations of the intensity of the envelope itself. (**c**) Superpixels were generated by removing dilated edges. (**d**) Small superpixels and those in contact with the image boundary were discarded and the remaining superpixels were smoothed and filled, before discarding those by size that did not belong to the nucleus. (**e**) Final segmentation of the NE overlaid (thick black line) on the filtered image. (**f**) Ground truth (GT) manual segmentation of the same image. Regions not selected by the algorithm because of their sizes are highlighted with arrows. Notice that both the algorithm and GT detected disjoint regions.

In this work, the NE of seven different cells were segmented and analysed. Intermediate steps of the segmentation algorithm consist of traditional image-processing steps: Low-pass filtering, edge detection, dilations, determination of superpixels, distance transforms, morphological operators, and post-processing to automatically segment the nuclear envelope (Figure 2). Images were initially low-pass filtered with a Gaussian kernel, with size *h* = 7 and standard deviation *σ* = 2, to remove high frequency noise. The algorithm exploited the abrupt change in intensity at the NE compared with the neighbouring cytoplasm and nucleoplasm by Canny edge detection [49]. The edges were dilated to connect disjoint edges by using the structural element of size 5 or greater than 5 depending on the standard deviation of the Canny edge detector. These disjoint edges were part of the NE and were initially missed due to intensity variations in the envelope itself (Figure 2b). The connected pixels not covered by the dilated edges were labelled by the standard 8-connected objects found in the image to create a series of superpixels (Figure 2c). We have dilated the same size as the width of the NE. The superpixel size was not restricted so that large superpixels covered the background and nucleoplasm. Morphological operators were used to remove regions in contact with the borders of the image, remove small regions, fill holes inside larger regions, and close the jagged edges (Figure 2d).

**Figure 3:**
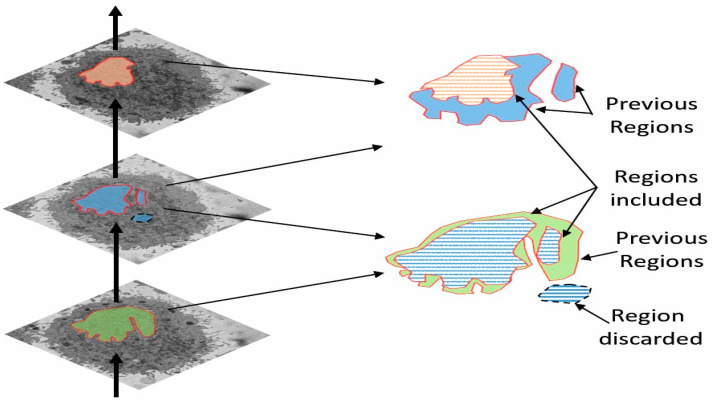
Illustration of the propagation regions. Regions of a slice that overlap with the regions of a previous segmentation are maintained, whilst regions that do not are discarded. The algorithm propagates up and down, starting from central slice (slice number 150 for all seven cells which were segmented in this work), in the same way as a human expert to include or discard regions.

A preliminary work approach described in [50] assumed that there was only one nuclear region in each image, which was designated as the nucleus, and any disjoint regions which could or not be part of the nucleus were ignored. That approach worked well for cells with a smooth near-spherical nuclear morphology. However, in cells with an irregular NE, disjoint regions were missed. To improve this approach, the new algorithm exploited the 3D nature of the data by using adjacent images to check for connectivity of islands to the main nuclear region, as a human operator would. It should be highlighted that the improvement is not only for small regions—these could be large. The original algorithm would only consider one region, and even if the second region would be close in area to the first one, it would be discarded. The algorithm thus began at the central slice of the cell, which was assumed to be the one in which the nuclear region would be centrally positioned and have the largest diameter. The algorithm assumed that this slice would only have one nuclear region. The algorithm then proceeded in both directions (up and down through the neighbouring slices) and propagated the region labelled as nucleus to decide if a disjoint nuclear region in the neighbouring slices (above or below) was connected above or below the current slice of analysis (Figure 3). When a segmented nuclear region overlapped with the previous nuclear segmentations, it was maintained; when there was no overlap, it was discarded.

By using neighbouring segmentations as input parameters to the current segmentation, and taking the regions into account, the algorithm was able to identify disjoint nuclear regions as part of a single nucleus (Figure 2e). Finally, the NE was obtained as the boundary of the nucleus. 

**Figure 4:**
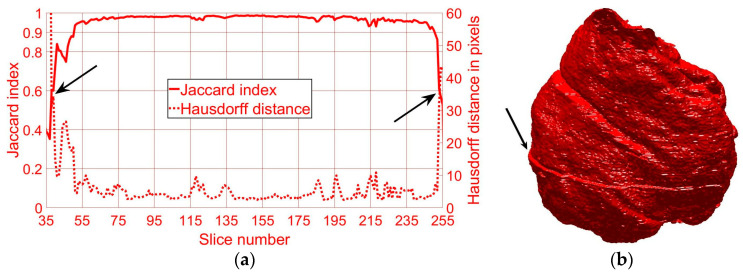

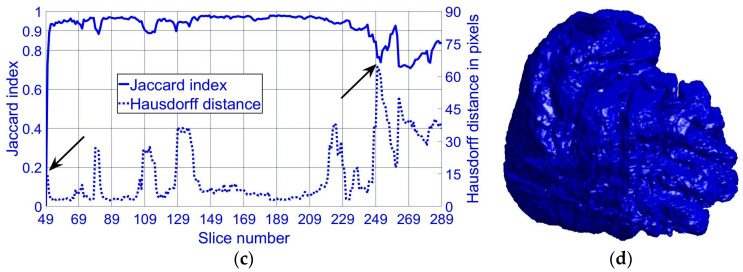
Similarity metrics and final results of the automated segmentation displayed as rendered volumetric surfaces. (**a**) Jaccard Index (JI) (solid line) and Hausdorff distance (HD) (dotted line) for the segmented nuclear envelope (NE) shown in (**b**). Notice that the mean JI is 98% and the mean HD is 4 pixels for central slices. (**b**) Final result of the segmentation displayed as a rendered volumetric surface. The algorithm was developed and validated with this cell, and the arrow indicates an artefact due to a slight shift in the data during the image acquisition. The artefact was corrected with a pre-alignment. (**c**) JI and HD for the second NE shown in (**d**). Note the decrease in JI and increase in HD (arrows) towards the upper and lower edges of the NE, where the structure tends to become more fragmented in two-dimensional (2D) cross-sections and complex regions that may have been missed by the algorithm similar to those highlighted in Figure 2f.

The algorithm was developed and validated on one cell (Figure 4b), and tested on the NEs of the other six cells of which only one had a corresponding GT with 300 slices (Figure 4d). All processing and visualisation was performed in MATLAB^®^ (The Mathworks^TM^, Natick, USA).

### 2.4. Quantitative Comparisons

**Figure 5:**
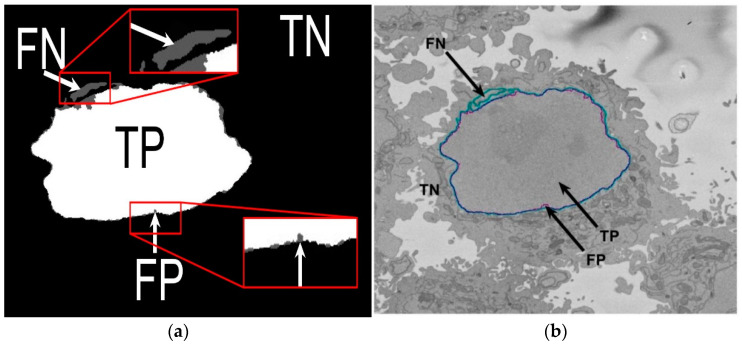
Illustration of the pixel-based metrics. (**a**) True Positives (TP, nuclear pixels segmented as nucleus), true negatives (TN, background pixels segmented as background), false positives (FP, background pixels segmented as nucleus), and false negatives (FN, nuclear pixels segmented as background). (**b**) Segmentation overlaid on one image: Ground truth (GT) in cyan, automated segmentation in purple.

In order to assess the accuracy of the segmentation, the pixel-based metric Jaccard similarity index (JI) [52] was calculated. JI is also known as intersection over union. This metric is based on the determination of the true positives (TP, nuclear pixels segmented as nucleus), true negatives (TN, background pixels segmented as background), false positives (FP, background pixels segmented as nucleus), and false negatives (FN, nuclear pixels segmented as background), as illustrated in Figure 5 and defined by the following Equation:

(1) $$\mathrm{Jaccard}\;\mathrm{Similarity}\;\mathrm{Index}\;=\;\frac{TP}{TP+FP+FN}$$

In addition, the Hausdorff distance (HD) of the maximum of the set of shortest distances between two curves was calculated to assess how far the real boundary of the NE was from the segmented NE.

HD [53] is defined as the maximum distance between a point on one curve and its nearest neighbour on the other curve [54,55] and defined by the following Equation:

(2) $$d_{H}\left(A,B\right)=max\left\{\begin{array}{c} sup\;inf\;d\left(a,b\right),\;sup\;inf\;d\left(a,b\right)\\ a\in A\;\;b\in B\;\;\;b\in B\;\;a\in A\; \end{array}\right\}$$

In both cases, the metrics (JI, HD) were calculated on a per-slice basis. JI is a strict measurement as compared with accuracy and other metrics, as it does not include the true negatives. On the other hand, accuracy includes the TN in both numerator and denominator and this, especially in cases where the objects of interest are small and there are large areas of background (e.g., the upper and lower slices of the cell), would render very high accuracy. As JI does not count TN, the values decrease towards the higher and lower slices of the cells, as can be seen in the edges of Figure 4a,c. Accuracy values would increase in these slices.

### 2.5. Active Contours

**Figure 6:**
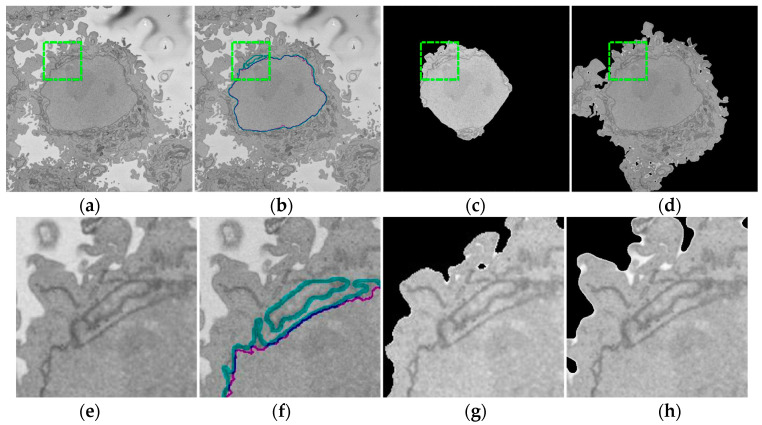
Comparison of the algorithm against ground truth (GT) and Active Contours. (**a**) One representative slice from one HeLa cell (red box in Figure 1b). Green dashed-dotted box denotes the region of interest (ROI) that is magnified in (**e–h**). (**b**) Automated segmentation of the nuclear envelope (NE) (purple). For comparison, the hand segmented GT is shown (cyan). (**c**) Active contours segmentation with 2200 iterations provided the highest Jaccard index (JI) 75%. (**d**) Active contours segmentation with 5000 iterations included large sections of the cell showing the influence of the iterations on the result.

In order to compare the algorithm segmentation with an alternative approach, the NE was segmented with Chan–Vese active contours methodology [56] (Figure 6). The function changes its parameters based on one of three states: Shrink, Grow, or Normal. One of the three states and its parameters were chosen empirically through numerous tests. A small circle was placed in the middle of the HeLa nucleus in one of the 300 images (Red box in Figure 1b) with the help of the MATLAB^®^ roipoly function and allowed to grow for a number of iterations. The number of iterations was increased in steps of 100 with the objective of determining the optimum number, i.e., to obtain the highest JI, and to continue until the algorithm had segmented not just the NE, but the whole cell. Practically, this consisted of running the algorithm up to 5000 iterations. In total, 50 out of 300 slices were segmented by the same method, and both JI and HD were computed. They were compared with the algorithm segmentation metrics. The parameters were adjusted (contraction bias = −0.4, smooth factor = 1.5, iterations = 5000) and the active contours was run again to obtain foreground and background.

Shrink and Normal, the other two states of active contours, were also implemented but the best result was obtained by the state of Grow.

### 2.6. Nuclear Envelope Shape Modelling 

**Figure 7:**
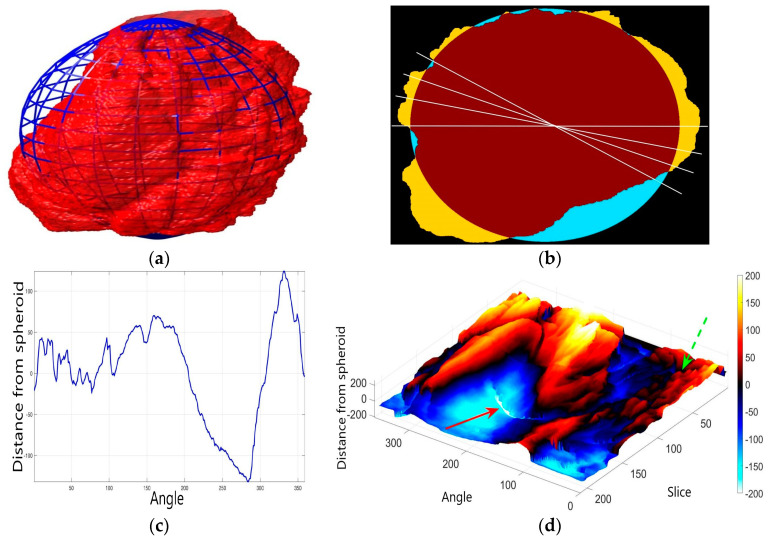
Nuclear envelope (NE) surface modelling against a spheroid. (**a**) Rendering of the nuclear envelope (NE) (red surface) against the model spheroid (blue mesh). (**b**) Illustration of distance calculations by ray tracing in one slice. Yellow regions correspond to the nucleus outside the spheroid, cyan regions to nucleus inside the spheroid. (**c**) Measurements obtained along the boundary. (**d**) Surface corresponding to the distance from the NE to a model spheroid. Solid red arrow indicates a notch, dashed green arrow shows rugged region.

In order to further study the shape of the segmented NE, this was modelled against a spheroid. The spheroid was created with the same volume as the nucleus and the position adjusted to fill the NE as closely as possible, as illustrated in Figure 7a, where NE is displayed as a red rendered volumetric surface and the spheroid as blue mesh

The surfaces of the spheroid and the nucleus were subsequently compared by tracing rays (Figure 7b) from the centre of the spheroid and the distance between the surfaces for each ray was calculated (Figure 7c). It was designated that when the NE was further away from the centre, the difference was positive. Figure 7d shows the surface corresponding to the distance from the NE of the first cell (Figure 4b) to a model spheroid. The notch that travels along the cell between slices is visible (red arrow) and the dashed green arrow highlights a particularly rugged region (Figure 7d). It should be noted that this process is automatic without any user intervention. In order to position the spheroid, the centroid of the segmented cell was calculated, and the coordinates were used as the centre of the spheroid.

Numerous metrics that characterise the NE can now be extracted, either directly from the NE: nuclear volume, Jaccard Index (JI) to the spheroid, or from the altitudes of the modelled surface: (mean value (μ) and standard deviation (σ)), range of altitudes. Other derived metrics can also be extracted; for instance, the ratio of the number above or below a certain threshold like the μ ± σ. Finally, the correlation between JI and statistical values were calculated so that some conclusions could be drawn about biological characteristics of cells that are beyond the scope of this article.

## 3. Results and Discussion

**Figure 8:**
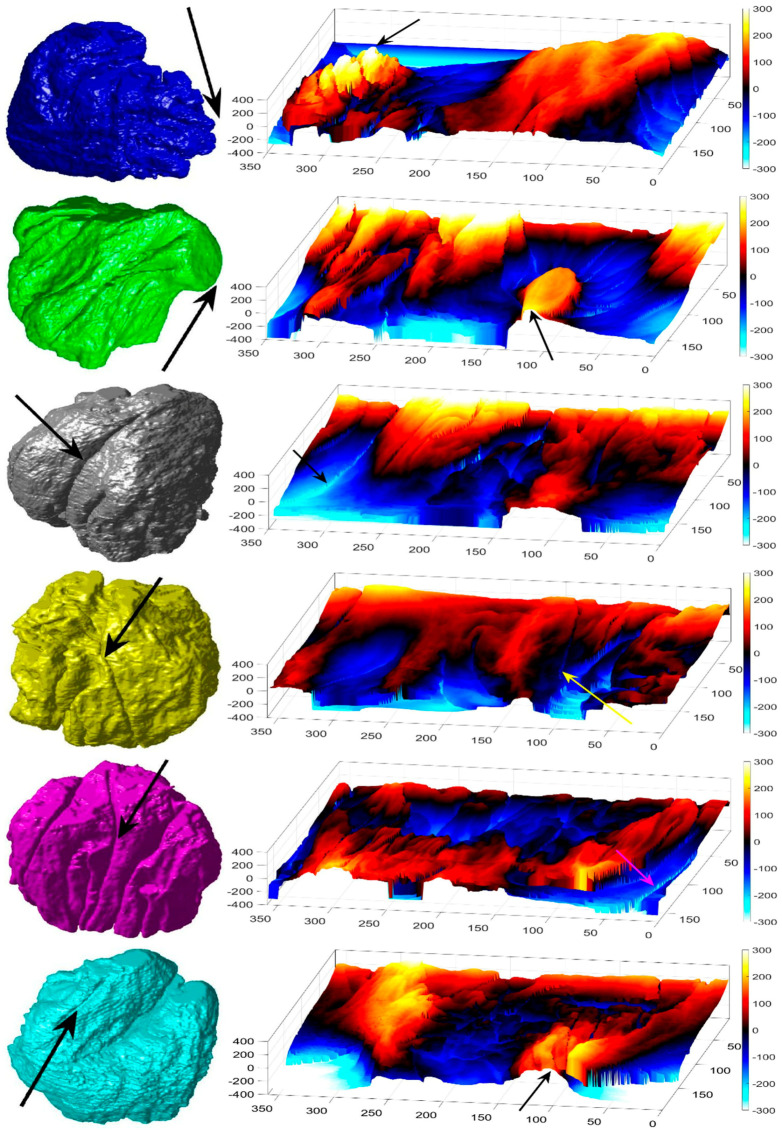
Final results of the automated segmentation displayed as rendered volumetric surfaces and modelling for six cells. Left: Segmentation results displayed as rendered volumetric surfaces for six different cells. In each cell, note the notches and invaginations, which may be relevant biological characteristics of the cells. Right: Surfaces corresponding to the distance from the nuclear envelope (NE) to a model spheroid. Arrows, in the last four rows, show notches that travel along the nuclei (grey, yellow, purple, and cyan cell).

The algorithm was designed and trained on one cell from which the parameters were derived. The algorithm was then applied to seven cells from which the nuclear envelope was segmented (Figure 4b,d and Figure 8 (Left)) with accurate results for the two sets for which 300 slices of ground truth were available and good visual assessment of the remaining five. The shapes of the final segmentations show the complexity of the NE with rather convoluted notches and invaginations. It is speculated that these shapes may have biological significance which is beyond the scope of this paper.

Whilst segmenting the first cell, a displacement artefact, which is assumed to have been caused by an external vibration to the microscope, was detected (arrow in Figure 4b). The slices were carefully observed by scrolling up and down, and a displacement was found between slices 121 and 122. A rigid registration algorithm was applied to measure the shift between slices, which reported a displacement of 11 rows and 20 columns. This displacement was corrected by shifting slices 122 to 300, and since the displacement was present on both images and GT, it had no impact on the JI or HD.

The automated segmentations were compared to manual GT segmentations in order to assess the accuracy of the algorithm. Two different similarity metrics, JI and HD, were computed. For the cell shown in Figure 4b (red cell), all 300 slices were visually checked and it was noticed that the bottom 26 and top 40 slices did not contain any cell—therefore, they were not included in similarity metrics calculations. The algorithm detected cells in all slices between 42 and 254, and JI and HD were computed as 93% and 9 pixels, respectively. For the slices between 50 and 250, the mean JI is 95% and the mean HD 8, and for slices between 75 and 225 (interquartile range, IQR), the mean JI is 98% with the mean HD 4 pixels (Figure 4a). Similarly, the bottom 40 of 300 slices of the cell shown in Figure 4d showed no cell 259 and the algorithm detected the cell for all slices between 47 and 289, and JI and HD were computed as 90% and 17 pixels, respectively. The mean JI is 93% and the mean HD 13 pixels for slices between 50 and 250, and 94% with the mean HD 13 pixels for slices between 75 and 225 (Figure 4c). JI decreased and HD increased towards the top and bottom of the cells, as the structure was considerably more complex and the areas become much smaller (Figure 4a,c).

Active contours, on the other hand, provided much lower values of JI with a highest value of 75% (illustrated on slice 118/300—Figure 6c,d), and took considerably longer, as 2200 iterations required 27 minutes for one slice only. In total, 50 out of 300 slices were segmented by active contours in 31 h. On the other hand, the algorithm described segmented each slice in ~8 s and the whole cell in approximately 40 min; that is, the segmentation of two slices with active contours would take longer than the algorithm for 300 slices.

The comparison between the model spheroid and the whole nucleus (shown in Figure 4b) reported a JI of 66% (Figure 7). Notice that in this case, JI is measuring how spherical the NE is, not the accuracy of the segmentation. This value indicates a relative departure from a spheroid and it is speculated that the JI could be related to biological characteristics of cells. In addition, the measurements of distance from the nucleus to the spheroid showed rougher and smoother regions (Figure 7a,b,d). The surface corresponding to the distance from the nuclear envelope to a model spheroid (Figure 7d) showed graphically the hollow and prominent regions of the cell, but more important, elements such as a notch (solid red arrow) or ruggedness (dashed green arrow) can be an indication of NE breaking down or remodelling. An advantage of this modelling is that visually it is easier to assess a single 2D image than a 3D volumetric surface, as can be seen in Figure 8 where six cells and their corresponding surfaces are shown. Mercator map projection was used in this modelling against a spheroid and this could be a limitation of the algorithm. Whilst there are many interesting characteristics such a large notch on the NE of the third (grey) cell or large protuberance on the second (green) cell, at this moment it is only possible to speculate the biological correlation between the surfaces and the nature of the cell itself.

Table 1 illustrates the previously described metrics that can be extracted from the NE and the surface. Mean (μ), standard deviation (σ), and range of values, which are related to the height of peaks and depth of valleys, could be related to some biological state of the cells; however, this has not yet been verified.

A strong negative correlation between JI and σ (correlation coefficient = −0.9139) is due to the similarity between the NE and the spheroid, as a NE which is closer to a spheroid (higher JI) will have a smoother surface (lower σ). This is also indicated by a weaker negative correlation between JI and range (correlation coefficient = −0.7388) and a positive correlation between JI and µ with correlation coefficient (0.7142).

## 4. Conclusions

An algorithm to segment and model the volumetric shape of the nuclear envelope of HeLa cells has been described. The NE of seven cells, 300 slices each, were successfully segmented, with good accuracy for the two data sets for which GT was available. It was observed that the segmentation accuracy was higher in the central slices than on the edges. This would be expected, as the NE is more irregular on the top and bottom slices than on the central ones. When the NE is modelled against a spheroid, it is possible to extract several quantitative metrics. We speculate that these metrics may be related to the biological characteristics of the cells. A biological comparison between different populations of cells is beyond the scope of this work, which focuses on the description of the segmentation and modelling of the NE. The segmentation of each cropped cell is fully automatic and unsupervised and segments one slice in approximately 8 s, and one whole cell in approximately 40 min. These results outperform a segmentation with Active Contours, both in terms of time and accuracy.

The main contributions of this work are: (a) An algorithm to segment the nuclear envelope of HeLa cells from SBF SEM images, which includes disjoint regions of the nucleus; (b) the modelling of the NE surface against spheroid, which could reveal interesting biological characteristics of the nucleus; and (c) the 2D maps of the NE surface, which can provide an easier way to assess the characteristics of a 3D structure.

The open-source algorithm described in this paper provides an alternative to expensive commercial software and manual segmentation, which is still widely used despite the significant disadvantages of time and inter- and intra-user variability.

## Figures and Tables

**Table 1:** Quantitative metrics extracted from surface modelling. Examples of metrics that can be automatically extracted from the nuclear envelope (NE) segmentation, including nuclear volume and Jaccard Index (JI) against the spheroid, and the mean value (μ), standard deviation (σ), range of values for the NE (distance of the highest peaks and deepest valleys from the spheroid). The last column shows the ratio of the number of pixels to the total number of pixels in one standard deviation above and below the mean value, as height/depth of a peak/valley may not indicate if this is a thin spike or more of a plateau. In addition to a strong negative correlation between JI and σ, with correlation coefficient (−0.9139) which explains the similarity between the NE and the spheroid, and a weaker negative correlation between JI and range, with correlation coefficient (−0.7388), a comparatively weaker positive correlation between JI and µ with correlation coefficient (0.7142) was observed. These values could be used to draw some conclusions about biological characteristics of cells and more metrics can be extracted from the algorithm developed for this work.

Cell	Volume Metrics		Surface Metrics				
3D Shape	Volume (μm^3^)	Jaccard Index	Surface Modelling against a Spheroid	Mean (μ)	Standard dev. (σ)	Range	Pixel Ratio for μ ± σ
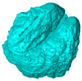	393	0.5538	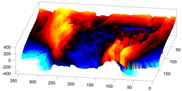	−23.424	142.47	681	16% & 19%
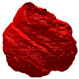	442	0.6610	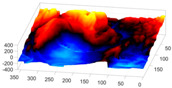	−17.018	105.11	517	13% & 18%
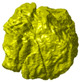	454	0.6989	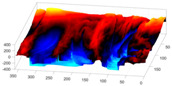	−11.013	96.968	553	12% & 17%
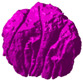	487	0.7084	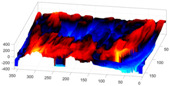	−16.467	98.528	577	15% & 15%
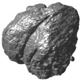	502	0.6643	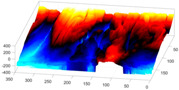	−27.290	116.11	544	13% & 19%
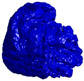	580	0.5991	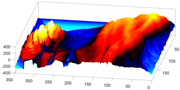	−27.882	135.57	703	19% & 18%
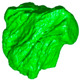	600	0.5801	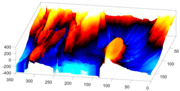	−29.894	163.80	894	17% & 15%

## Supplementary Materials

The code associated with this article is available open source at https://github.com/reyesaldasoro/Hela-Cell-Segmentation, and the data are available at: http://dx.doi.org/10.6019/EMPIAR-10094.

## Abbreviations

EM	Electron Microscopy
GT	Ground Truth
HD	Hausdorff Distance
JI	Jaccard Index
NCMIR	National Centre for Microscopy and Imaging Research
NE	Nuclear Envelope
ROI	Region of Interest
SBF SEM	Scanning Block Face Electron Microscopy
TIFF	Tagged Image File Format
